# p53 regulates ERK1/2/CREB cascade *via* a novel SASH1/MAP2K2 crosstalk to induce hyperpigmentation

**DOI:** 10.1111/jcmm.13168

**Published:** 2017-04-06

**Authors:** Ding'an Zhou, Zhongshu Kuang, Xing Zeng, Ke Wang, Jiangshu Ma, Huangchao Luo, Mei Chen, Yan Li, Jiawei Zeng, Shu Li, Fujun Luan, Yong He, Hongying Dai, Beizhong Liu, Hui Li, Lin He, Qinghe Xing

**Affiliations:** ^1^ Clinical Research Center Affiliated Hospital of Guizhou Medical University Guiyang Guizhou China; ^2^ Yongchuan Hospital Chongqing Medical University Chongqing China; ^3^ Children's Hospital and Institutes of Biomedical Sciences Fudan University Shanghai China; ^4^ Dujiangyan People's Hospital Cheng du Sichuan China; ^5^ Department of Nephrology and Rheumatology the First People's Hospital Chenzhou Hunan China; ^6^ Bio‐X Institute Key Laboratory for the Genetics of Developmental and Neuropsychiatric Disorders (Ministry of Education) Shanghai Jiao Tong University Shanghai China

**Keywords:** SASH1‐MAP2K2 crosstalk, p53‐POMC‐MC1R cascade, ERK1/2/CREB cascade, hyperpigmentation

## Abstract

We previously reported that three point mutations in SASH1 and mutated SASH1 promote melanocyte migration in dyschromatosis universalis hereditaria (DUH) and a novel p53/POMC/Gαs/SASH1 autoregulatory positive feedback loop is regulated by SASH1 mutations to induce pathological hyperpigmentation phenotype. However, the underlying mechanism of molecular regulation to cause this hyperpigmentation disorder still remains unclear. In this study, we aimed to investigate the molecular mechanism undergirding hyperpigmentation in the dyschromatosis disorder. Our results revealed that SASH1 binds with MAP2K2 and is induced by p53‐POMC‐MC1R signal cascade to enhance the phosphorylation level of ERK1/2 and CREB. Moreover, increase in phosphorylated ERK1/2 and CREB levels and melanogenesis‐specific molecules is induced by mutated SASH1 alleles. Together, our results suggest that a novel SASH1/MAP2K2 crosstalk connects ERK1/2/CREB cascade with p53‐POMC‐MC1R cascade to cause hyperpigmentation phenotype of DUH.

## Introduction

Melanocytes are epidermal cells of neurocrest origin that synthesize melanin and are responsible for skin pigmentation and protection from ultraviolet (UV) radiation 1, 2. Melanocortin receptor 1 (MC1R), a G protein‐coupled receptor (GPCR) coupled to the α subunit of G protein, induces elevated concentrations of intracellular cyclic adenosine monophosphate (cAMP) upon binding a ligand such as α‐melanocyte‐stimulating hormone (α‐MSH). Adenosine 3′,‐5′‐cyclic monophosphate (cyclic AMP, cAMP), an important second messenger in melanogenesis, contributes to both melanin synthesis and movement of melanosomes to newly formed dendrites at the cell periphery 1, 2. MC1R, preferentially expressed in epidermal melanocytes 3, is activated by its ligand, α‐MSH, a pigmentation hormone produced and secreted by keratinocytes and melanocytes in the skin following UV exposure. Upon ligand binding, GPCRs impart a signal to heterotrimeric G proteins composed of α, β and γ subunits, leading to the dissociation of the Gα subunit from the Gβγ subunit. These activated G proteins of the guanine nucleotide‐binding protein subunit alpha isoforms short (Gαs) class directly catalyse the conversion of adenosine triphosphate (ATP) to cAMP 4.

*SASH1*, originally identified as a candidate tumour suppressor gene in breast and colon cancer, regulates the adhesive and migratory behaviours of cancer cells during tumour formation. Functional domain analysis has demonstrated that SAM and SH3 domain containing 1 (SASH1) protein may belong to the previously described novel family of putative adapter and scaffold proteins that transfers signals from ligand–receptor interactions 5, 6, 7. Our previous study indicated that SASH1 is associated with Gαs downstream of α‐MSH/MC1R signalling cascade 8. Our recent study also revealed that SASH1 is regulated by a novel p53/POMC/α‐MSH/Gαs/SASH1 cascade to mediate melanogenesis. A novel p53/POMC/Gαs/SASH1 autoregulatory positive feedback loop is regulated by SASH1 mutations to induce pathological hyperpigmentation phenotype 9. However, whether ERK1/2 signal pathway which mediates melanogenesis 10, 11 and microphthalmia transcription factor (Mitf), a melanocyte master transcription factor which is also responsible for pigment cell‐specific transcription of the melanogenesis enzyme genes 12, are mediated by SASH1 mutations remains unknown.

P53, a canonical transcriptional factor, has been suggested to directly activate transcription of numerous genes such as those that control cell cycle, apoptosis and others. P53 directly mediates UV induction of POMC‐MSH signal cascade in skin and stimulates the POMC promoter in response to UV and is involved in UV‐independent pathologic pigmentation and could mimic the tanning response 13. Based on our previous report that SASH1 binds with Gαs,the downstream molecule of POMC/MSH‐MC1R signalling cascade 8, we deduce that SASH1 may be mediated by p53‐POMC‐MC1R cascade.

Based on the aforementioned findings, we hypothesize that SASH1 may be mediated by the p53‐POMC‐MC1R cascade and crosstalks with ERK1/2 pathway to regulate melanogenesis. Herein, our work shows that p53‐α‐MSH/POMC‐MC1R‐Gαs cascade could transactivate ERK1/2/CREB cascade *via* a novel SASH1/MAP2K2 crosstalk to cause hyperpigmentation of dyschromatosis universalis hereditaria (DUH).

## Materials and methods

### Main reagents and cell lines

[Nle^4^,D‐Phe^7^]‐α‐MSH(NDP‐MSH) was obtained from Tocris Bioscience (R&D Systems, Minnesota, USA). Forskolin (FSK) was purchased from Beyotime Biotechnology, Inc (Jiangsu, China). 8‐Bromo‐cAMP (sc‐201564) was obtained from Santa Cruz Biotechnology, Inc (California, American). SiRNAs were synthesized by Shanghai GenePharma Co., Ltd (Shanghai, China). The specific and efficient siRNA sequences of SASH1, CREB and MAP2K2 were as follows: SASH1 siRNA1 sequence: 5′‐GCACGUUCAAGUUCAUCUATT‐3′ (sense), 5′‐UAGAUG AACUUGAACGUGCTT‐3′ (antisense); SASH1 siRNA2 sequence: 5′‐GACUCAGGAU GCUACGAAATT‐3′ (sense), 5′‐UUUCGUAGCA UCCUGAGUCTT‐3′ (antisense); CREB siRNA1 sequence: 5′‐GCAGACAGUUCAAGUCCAUTT‐3′ (sense), 5′‐AUGGACUU GAA CUGUCUGCTT‐3′ (antisense); CREB siRNA2 sequence: 5′‐GCGAAGGGAA AUUCUUUCATT ‐3′ (sense), 5′‐UGAAAGAAUUUCCCUUCGCTT‐3′ (antisense); MAP2K2 siRNA1 sequence: 5′‐GCAUUUGCAUGGAACACAUTT‐3′ (sense), 5′‐AUGUGUUCCAUGCAAAUGCTT‐3′ (antisense); MAP2K2 siRNA2 sequence: 5′‐CUCCUGGACUAUAUUGUGATT‐3′ (sense), 5′‐UCACAAUAUAGUCCAGGAGTT‐3′ (antisense).

### Construction of SASH1, POMC, MAP2K2, MC1R and p53 expression vectors

Wild‐type (WT) and mutant SASH1‐pEGFP‐C3 and WT and mutant SASH1‐PBABE‐Flag‐puro constructs were created as per our previous study 8. To construct HA‐Pcna3.0‐p53, HA‐Pcna3.0‐MAP2K2, HA‐Pcna3.0‐MC1R and myc‐Pcdna3.0‐POMC vectors, PCR reactions of bacteria (obtained from the Han Jiahuai Lab, Xiamen University, Xiamen, China) containing the vector with full‐length CDS sequences of p53, MC1R, MAP2K2 and POMC were performed with GXL polymerase (Takara, Shimogyo‐ku, Kyoto, Japan) and the following primers: p53 primers 5′‐CGCGGA TCCGCCACCACCATGGAGGAGCCGCAGTCAGATCCTA‐3′ (forward, *BamH*I site included) and 5′‐CCGCTCGAGTCAGTCTGAGTCAGGCCCTTCTGTCTTGAACATGAGTTT‐3′ (reverse, *Xho*I site included); *MC1R* primers 5′‐CCCAAGCTTGCCACCGCC ACCATGGCT GTGCAGGGATCC‐3′ (forward, *Hind*III site included) and 5′‐CCGCTCG AGTCACCAGGAGCATGTCAGCA (reverse, *Xho*I site included); *MAP2K2* primers 5′‐CGGAATTCGCCACCGCCACCATGCTGGCCCGGAGGAAGCCG‐3′(forward, *ECoR*I site included) and 5′‐CCCTCGAGTCACACGGCGGT GCGCGTGGGTGTG‐3′ (reverse, *Xho*I site included); *POMC* primers 5′‐CGCGGATCCATGCCGAGATCGTGCTGC‐3′ (forward, *BamH*I site included) and 5′‐CCCAAGCTTTCACTCGCCCTT CTTGT AGGCG TTCTTGAT‐3′ (reverse, *Xho*I site included). To construct GFP wild‐type (WT) and mutant SASH1 constructs for transfection or infection, WT SASH1‐pEGFP‐C3 recombined vector (PCR template) and *SASH1* full‐length primers 5′‐ATAAGAAT GCGGCCGC ATGGAGGACGCGGGAGCA‐3′ (forward, *Not*I site included) and 5′‐CCGCTC GAGCTACATGGCCTCAGGGCCTGGCGGCAGTT‐3′ (reverse, *Hind*III site included) were used to amplify *SASH1* full length and ligate into pCDH‐EF1‐MCS‐T2A‐copGFP vector (CD521A‐1; System Biosciences, Shanghai, China). Restriction sites of all mammalian expression vectors were sequenced to verify correctly sized inserts in the proper orientation.

### Construction of SASH1 deletion constructs

The construction of deleted SASH1 construct plasmids including SASH1‐SH3‐SAM‐pEGFP‐C3 vector, SASH1‐ΔC‐terminal‐pEGFP‐C3 vector,SASH1‐ΔN‐terminal‐pEGFP‐C3 vector and SASH1‐C‐terminal‐pEGFP‐C3 vector was partly referred to the previous report 7. The 768‐bp central fragment of SASH1‐SH3‐SAM corresponding to the Sly‐homology domain, the 1743‐bp‐SASH1‐ΔC‐terminal fragment (SASH1_1‐581aa), the 1974‐bp‐SASH1‐ΔN‐terminal fragment (SASH1_590‐1247aa) and the 1617‐bp‐SASH1‐C‐terminal fragment (SASH1_709‐1247aa) were created by PCR with the SASH1 full‐length pEGFP‐C3 plasmid as template. The amplification primers used in PCR were as follows: SASH1‐SH3‐SAM‐fragment 5′‐CCGCTCGAGCTTGGGAAAAAGGTGAAATCAGTGA‐3′ (forward, XhoI site included), 5′‐GGGGTACCGTCGCTGTTACTGTCATACTCT‐3′ (reverse, KpnI site included); SASH1‐ΔC‐terminal fragment 5′‐CCGCTCGAGATGGAG GACGCGGGA GCAGCTGGCC‐3′ (forward, XhoI site included), 5′‐GGGGTACCATCTCCTTTCTTGAGC TTGAGTGAG‐3′ (reverse, KpnI site included); SASH1‐ΔN‐terminal fragment 5′‐CCGCTCGAG CCCATGGGGACCTGGATGGGCCTGC‐3′ (forward, XhoI site included), 5′ ‐GGGGTACCCTACATGGCCTCAGGGCCTGGCGG‐3′ (reverse, KpnI site included); SASH1‐C‐terminal fragment 5′‐CCGCTCGAGAAGCTGCTCGTTGACAGCCAG GGCC‐3′ (forward, XhoI site included),5′‐GGGGTACCCTACATGGCCTCAGGGCCTGGCGGC‐3′ (reverse, KpnI site included). The amplified fragments were purified and digested with XhoI and KpnI to subclone in pEGFP‐C3 vector (Clontech, Heidelberg, Germany). Restriction sites of the fragmented SASH1‐pEGFP‐C3 recombined vectors were sequenced to verify correctly sized inserts in the proper orientation.

### Cell culture and transfection

A375 cells and HEK‐293T cells (from Cell Bank of Shanghai Institute of Life Sciences, Chinese Academy of Sciences Shanghai, China) were identified by short tandem repeat (STR) detections and mycoplasma tests and cultured as described previously 14. Normal human primary melanocytes (NHEMs) were obtained from Promocell, Heidelberg, Germany (NHEM‐c M2/NHEM‐p M2, Order Number: C‐12402/C‐12452, Lot Number: 5120704.3) and cultured in human melanocyte growth medium (M2, Promocell). SASH1‐pCDH‐EF1‐MCS‐T2A‐copGFP vector infections were carried out with human melanocytes in 6‐cm dishes or 10‐cm dishes at a density of 200,000–50,000 cells/dish on 3 subsequent days. A375 and HEK‐293T cells were transfected using Entranster‐D (Engreen Biosystem Co., Ltd., Beijing, China), polyethyleneimine (PEI) or Lipofectamine^®^ 2000 (Thermo Fisher Scientific Inc., Rockford, IL, USA) as previously described 14, 15. Transfected A375 cells were cultured in 1.5 μg/ml puromycin to select stable cell lines. NHEMs and HEK‐293T cells were transiently transfected or infected with HA‐Pcna3.0‐p53,HA‐Pcna3.0‐MC1R and myc‐Pcdna3.0‐POMC vectors and SASH1‐pEGFP‐C3 or SASH1‐pCDH‐EF1‐MCS‐T2A‐copGFP recombined vectors to analyse expression of exogenous p53, POMC, MC1R and SASH1 using PEI or PromoFectin (Promocell) or packaged SASH1‐pCDH‐EF1‐MCS‐T2A‐copGFP virus. Transfected NHEMs and transfected HEK‐293T cells were introduced with specific siRNAs of SASH1, MAP2K2 and CREB to detect the effects of SASH silence, MAP2K2 and CREB on downstream.

### Pull‐down assay and nanoflow LC‐MS/MS and bioinformatic analyses

The protocols for the pull‐down assay, nanoflow LC‐MS/MS, database searches and bioinformatics analysis for functional classification are referred to our previous report 8.

### Immunoprecipitation (IP) and Western blot (WB)

HEK‐293T cells were gently washed in PBS for three times and then lysed for 20 min. using IP‐Western blot lysis buffer (P0013; Beyotime Biotechnology, Inc.) in the presence of a complete protease inhibitor cocktail, 1 μM sodium orthovanadate and 1 mM sodium fluoride per 6‐cm dish on ice. Cell lysates were transferred into 1.5‐ml microcentrifuge tubes. Extracts were centrifuged for 10 min. at 14007g at 4°C. Then, 300 μl of supernatants were pre‐cleaned with 20 μl of Protein A/G PLUS‐Agarose (sc‐2003; Santa Cruz Biotechnology, Inc.) for 1 hr and immunoprecipitated using 2 μl of DYKDDDDK Flag‐tag mouse mAb (M20008; Shanghai Abmart, Shanghai, China) or GFP‐Tag mouse Ab (E022030‐01; EarthOx, LLC, San Francisco, CA, USA) at 4°C for 2 hrs and mixed with 20 μl of Protein A/G PLUS‐Agarose (sc‐2003, Santa Cruz Biotechnology, Inc.) at 4°C for 8 hrs to perform immunoprecipitation assay. The immunoprecipitates were washed with PBS three times and subjected to SDS‐PAGE and Western blotting. The primary antibodies used in IP‐WB analysis were DYKDDDDK Flag‐tag mouse mAb (M20008; Shanghai Genomics, Shanghai, China), HA‐tag mouse mAb (SG4110‐25, Shanghai Genomics), SASH1 rabbit pAb (A302‐265A‐1; Bethyl Laboratories, Inc. Montgomery, Texas, USA) MAP2K2(MEK1/2) (D1A5) rabbit mAb(#8727; Cell Signaling Technology, Inc, Danvers, MA, USA), β‐tubulin mouse Ab (E022030‐01; EarthOx, LLC) and GAPDH mouse mAb (M20005; Shanghai Abmart, Inc.). HEK293T transfected cells and NHEMs were gently washed in PBS for three times and then lysed for 20 min. using IP‐Western blot lysis buffer. Cell lysates were transferred into 1.5‐ml microcentrifuge tubes. Extracts were centrifuged for 10 min. at 12,000 r.p.m. at 4°C. The primary antibodies used in the Western blot experiments were showed in Table S3. Immunoblotting was performed as previously described 16.

### Immunohistochemical and melanin staining

#### Immunohistochemical staining

Written informed consent regarding tissues and data used for scientific purposes was obtained from all participating patients. The study was approved by the Research Ethics Committees of Institutes of Biomedical Sciences, Fudan University, Shanghai, China, and Yongchuan Hospital, Chongqing Medical University, Chongqing, China. The fresh human foreskin tissues from a 14‐years‐old boy were fixed, pulled expansion and spread on a foam board and irradiated for different time length to reach 0.01, 0.05, 0.10, 0.50, 1.00 and 2.00 J/cm^2^, six doses of UVB (wavelength: 311 nm). After irradiation, these irradiated foreskin tissues and normal control (NC) and affected skin epithelial tissues from Y551D‐SASH1‐mutation individuals were fixed in 10% formalin at 4°C for 24 hrs and embedded in paraffin for immunohistochemistry analyses. The sections covered with foreskin tissues and skin epithelial tissues were deparaffinized and rehydrated using xylene and an ethanol gradient. Sections were incubated with p53 monoclonal antibody(mAb), ACTH polyclonal antibody (pAb), SASH1 pAb, phospho‐ERK1/2 mAb, phosphor‐CREB(Ser133) mAb, Mitf pAb, SILV mAb, tyrosinase pAb and TYRP1 mAb as well as horseradish peroxidase‐linked anti‐rabbit and anti‐mouse universal secondary antibodies. The detailed usage information of these antibodies is shown in Table S3. Finally, sections were counterstained with haematoxylin and photographed under the positive position microscope BX51 (Olympus, Beijing, China). The staining intensity and percentage of positive cells were calculated and scored in sections of each UV irradiation doses. The staining intensity of p53‐, ACTH‐, SASH1‐, p‐ERK1/2‐, p‐CREB‐, Mitf‐, Pmel17‐, TYRP1‐, SILV‐, tyrosinase‐positive cells was divided into four grades as ‘negative (−)’, ‘+’, ‘++’ and ‘+++’and calculated as 0, 1, 2 and 3 score, respectively. The positive cells’ percentage was divided into six categories as ‘negative (−)’, ‘1–20%’, ‘21–40%’, ‘41–60%’, ‘61–80%’ and ‘81–100%’ and calculated as 0, 1, 2, 3, 4, 5 and 6 score,respectively. Total scores of every visual field were determined by the formula

$$\begin{array}{c} \text{Staining intensity scores of positive cells}\\ \times \text{scores of positive}\;{\text{cells}}^{\prime }\;\text{percentage}\\ =\text{total scores of each view fields.} \end{array}$$

#### Melanin staining

Paraffin sections (5 μm) from normal and affected epithelial tissues were baked at 80°C for 30 min. and then kept at room temperature for 15 min. The tissue slices were incubated in GENMED dewaxing liquid (GenMed Scientifics Inc., Shanghai, China) for three times (15 min./time), GENMED dehydrated liquid for 3 min., GENMED strengthening liquid, GENMED clear liquid for 3 min. and GENMED cleaning liquid for 3 min. successively. Then, the GENMED cleaning liquid was removed and replaced with GENMED dyeing working fluid for about 30 min. at 45°C until dark brown of tissue slices appeared. GENMED dyeing working fluid was then removed and replaced with GENMED cleaning liquid for 2‐min. incubation,and GENMED cleaning liquid was removed and replaced with GENMED equilibrium liquid for 3‐min. incubation. GENMED equilibrium liquid was removed and replaced with GENMED cleaning liquid for 3‐min. incubation. Finally, the cleaning liquid was removed and incubated with GENMED stabilizing buffer for 2 min. GENMED stabilizing buffer was removed from the tissue slices, and tissue slices were washed with GENMED cleaning liquid at room temperature for 2 min. GENMED cleaning liquid was removed, and transparent processing of tissue slices was implemented, and tissue slices were mounted with neutral resin and photographed under the positive position microscope BX51 (Olympus). The detailed information of melanin staining reagents was as described as the manufacturer's protocol suggested.

### Quantification of intracellular cAMP levels

To identify the NHEMs (purchased from Promocell) and the HEK‐293T cells that were transfected HA‐MC1R‐pcdna3.0 vector beforehand have MC1R responsiveness, NHEMs and HEK‐293T transfected cells on 6‐well plates with four groups of repetition were starved for 8 hrs and treated with 10^−5^M of NDP‐MSH for 30 min., respectively. HEK293T transfected cells and NHEMs were diluted with 0.1 M HCL and incubated at room temperature for 20 min. and scraped with a cell scraper. After homogeneous bleeding by pipetting up and down, cell lysates were transferred into a 1.5‐ml centrifuge tube and centrifuged for 10 min. Protein concentrations of HEK293T transfected cells and NHEMs were determined by BCA kit (Beyotime Biotechnology Inc.) with bovine serum albumin (BSA) as a standard. Cell samples and standard cAMP stock were treated with acetylating reagent. The detailed subsequent procedures for detection of cAMP levels and producing a standard curve were as per the manufacturer's protocol (cAMP Direct Immunoassay Kit; BioVision Shanghai, China). Finally, absorbance readings were determined at OD_450_, and relative cAMP levels in HEK‐293T cells and NHEMs were calculated.

#### Tyrosinase activity and melanin content assays

##### Tyrosinase activity analysis

NHEMs transfected or infected with WT and mutant SASH1 or transfected or infected NHEMs silenced by MAP2K2 and CREB siRNAs were washed with GenMed cleaning liquid provided by the manufacturer (GenMed Scientifics Inc.), lysed with GenMed lysis buffer and centrifuged at 16,000 × *g* for 5 min. to obtain the supernatant for tyrosinase activity and protein concentration analyses. The protein concentration was determined with Bradford protein concentration quantification kit (GenMed Scientifics), and the remaining supernatant was placed on the ice for tyrosinase activity analyses. Based on the principle that dopachrome can be generated by tyrosine under the catalysis of tyrosinase, activity of tyrosinase can be determined by the changes in dopachrome absorbance at 475 nm wavelength. GENMED buffer and GENMED substrate solution were mixed and filled with oxygen for 2 min. Negative control liquid and 50 μl of remaining supernatant were added into the mixed solution and mixed with up‐down blending for background control measurement and sample tyrosinase activity determination, and the mixture was incubated at room temperature for 60 min. The absorbance of negative control liquid and samples was measured at 475 nm wavelength. The tyrosinase activity of samples was determined by the formula

$$\begin{array}{cc} & \left[\right.\left(\text{sample absorbance ‐ background control absorbance}\right)\\ & \;\times \text{Sample dilution ratio}*0.25\left(\text{System volume, ml}\right)]\\ & \;÷\left[\right.0.01\left(\text{sample volume, ml}\right)\\ & \;*3.6(\text{Millimolarabsorption coefficient})\\ & \;*60\left(\text{reaction time, min.}\right)]\\ & \;=\text{micromole dppachrome/min. ml}\\ & \;÷(\text{sample protein concentrationmg/ml})\\ & \;=\text{micromole dppachrome/min. mg.} \end{array}$$

##### Melanin content assays

NHEMs infected with WT and mutant SASH1 were washed with GenMed cleaning liquid provided by the manufacturer (GenMed Scientifics Inc.), lysed with GenMed lysis buffer at 4°C for 30 min. and proceeded with 16,000 × *g* centrifugation for 5 min. at 4°C. The cell lysates of NHEMs were centrifuged at 16,000 × *g* for 5 min. at 4°C, and 10 μl of supernatant was implemented to protein concentration determination. 500 μl of GENMED treating fluid was added to the remaining supernatant and centrifuged at 16,000 × *g* for 5 min. The supernatant was removed, and 500 μl of GENMED dissolving solution was added into cell deposits and mixed. 500 μl of GENMED buffer was added and stored at 60°C for 30 min. avoiding exposure. The melanin content was determined by measuring the absorbance at 360 nm wavelength.

Meanwhile, 500 μl of GENMED buffer was added to five numbered Eppendorf centrifuge tubes and 500 μl of GENMED melanin standard solution was added to No.1 Eppendorf centrifuge tube and mixed. 500 μl of mixture transferred from No.1 Eppendorf centrifuge tube was added into No.2 centrifuge tube. And the remaining tubes were added in turn. Finally, the melanin standard solution was also added with 500 μl of GENMED dissolving solution and GENMED buffer and incubated at 60°C for 30 min. The absorbance of standard melanin content at 360 nm wavelength was determined, and the standard curve was drawn to analyse the melanin content of samples.

### Statistical analyses

The data are presented as mean ± standard derivation (S.D.). These data were first analysed using the homogeneity of variance test, followed by the change of variable test. Statistical significance was determined by a one‐factor analysis of variance (anova) using LSD correction on (IBM (International Business Machine), Shanghai, China) to generate the required *P*‐values. Cartograms were plotted using GraphPad Prism 5 (GraphPad Software, Inc.La Jolla, CA, USA).

## Results

### SASH1 is induced by the p53/POMC cascade

**Figure 1:**
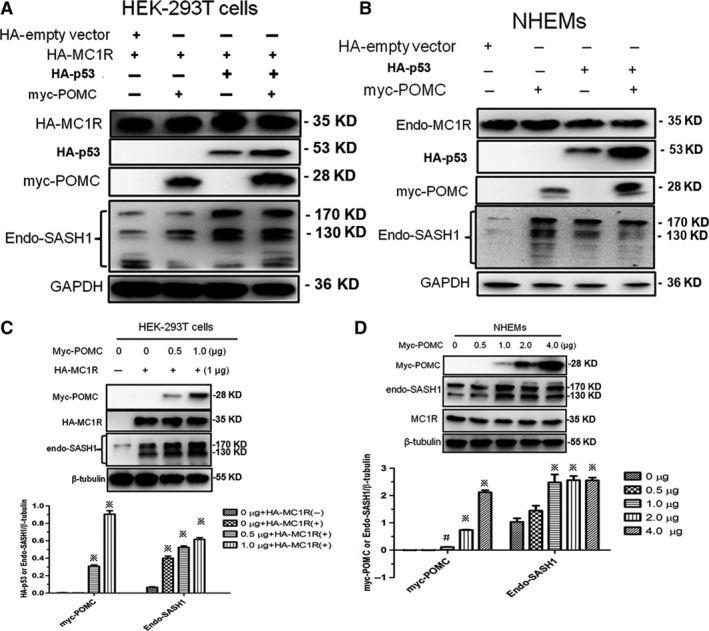
SASH1 is induced by the p53‐POMC cascade. (**A**) and (**B**) Increased SASH1 was induced by exogenous p53 (HA‐p53) and/or exogenous POMC (myc‐POMC) in HEK‐293T transfected cells and NHEMs. HA‐MC1R was beforehand transfected into HEK‐293T cells. At 24 hrs after transfection, HA‐p53 and myc‐POMC were transfected into HEK‐293T transfected cells and NHEMs, respectively. At 48 hrs after transfection, cells were lysed and subjected to immunoblot with GAPDH as loading control. (**C**) Exogenous POMC (myc‐POMC) triggers endogenous SASH1 expression in a dose‐dependent manner. Increased doses of myc‐POMC plasmid were introduced into HEK‐293T cells with HA‐MC1R pre‐transfection or without HA‐MC1R pre‐transfection. At 48 hrs after transfection, with β‐tubulin as a loading control, immunoblotting was performed to assess the effects of myc‐POMC on endogenous SASH1 in the presence or absence of HA‐MC1R. Endogenous SASH1 was induced by gradually increased dose of myc‐POMC in the presence of exogenous MC1R rather than the absence of exogenous MC1R. Top panel: cell lysates were analysed by Western blot using specific antibodies. Bottom panels: relative protein levels of myc‐POMC and endogenous SASH1 were determined by Western blot in three independent experiments. ※ *P* < 0.01 compared to 0 μg +HA‐MC1R(−) group and 0 μg +HA‐MC1R(+) group. (**D**) POMC triggers SASH1 expression in the presence of endogenous MC1R. Increasing amounts of myc‐POMC plasmid were introduced into NHEMs. Exogenous POMC and endogenous SASH1 were measured by Western blot and normalized to β‐tubulin. Top panel: Western blot results using specific antibodies. Bottom panels: relative protein levels of myc‐POMC and endogenous SASH1 were determined by Western blot in three independent experiments. #*P* <0.05 compared to 0 μg group, ※ *P* < 0.01 compared to 0 μg group. NHEMs: normal human epithelial melanocytes; SASH1: SAM and SH3 domain containing 1.

Our previous study demonstrated that SASH1 protein binds to Gαs 8. Moreover, it has been shown that Gαs, a major activator of adenylyl cyclase and cAMP production in melanocytes, is stimulated by α‐MSH and melanocortins 17. α‐MSH and other bioactive peptides are identified to be the cleavage products of pro‐opiomelanocortin (POMC), a multicomponent precursor for α‐MSH (melanotropic hormone), ACTH (adrenocorticotropic hormone) and the opioid peptide β‐endorphin. Normal synthesis of α‐MSH and ACTH is an important determinant of constitutive pigmentation in human beings and the cutaneous response to UV 3. P53 directly stimulates the POMC promoter in response to UV and also induces UV‐independent pathologic pigmentation 13. Hence, to determine whether SASH1 is mediated by p53 and POMC, the MC1R ligand, exogenous p53 and exogenous POMC were introduced into HEK‐293T cells pre‐transfected with exogenous MC1R and NHEMs to investigate the effects of MC1R ligands on SASH1. Endogenous SASH1 was found to be induced by introduction of exogenous p53 and POMC in NHEMs and HEK‐293T transfected cells (Fig. 1A and B).

We also investigated SASH1 responsiveness to POMC inducement. Exogenous POMC was introduced into NHEMs and HEK‐293T transfected cells. POMC was shown to increase expression of endogenous SASH1 (Fig. 1C and D). As documented in Figure S1, NHEMs and HEK‐293T transfected cells were stimulated by NDP‐MSH to secrete more intracellular c AMP as compared to that of DMSO control, which indicated that NHEMs and the transfected HEK‐293T cells had MC1R responsiveness.

**Figure 2:**
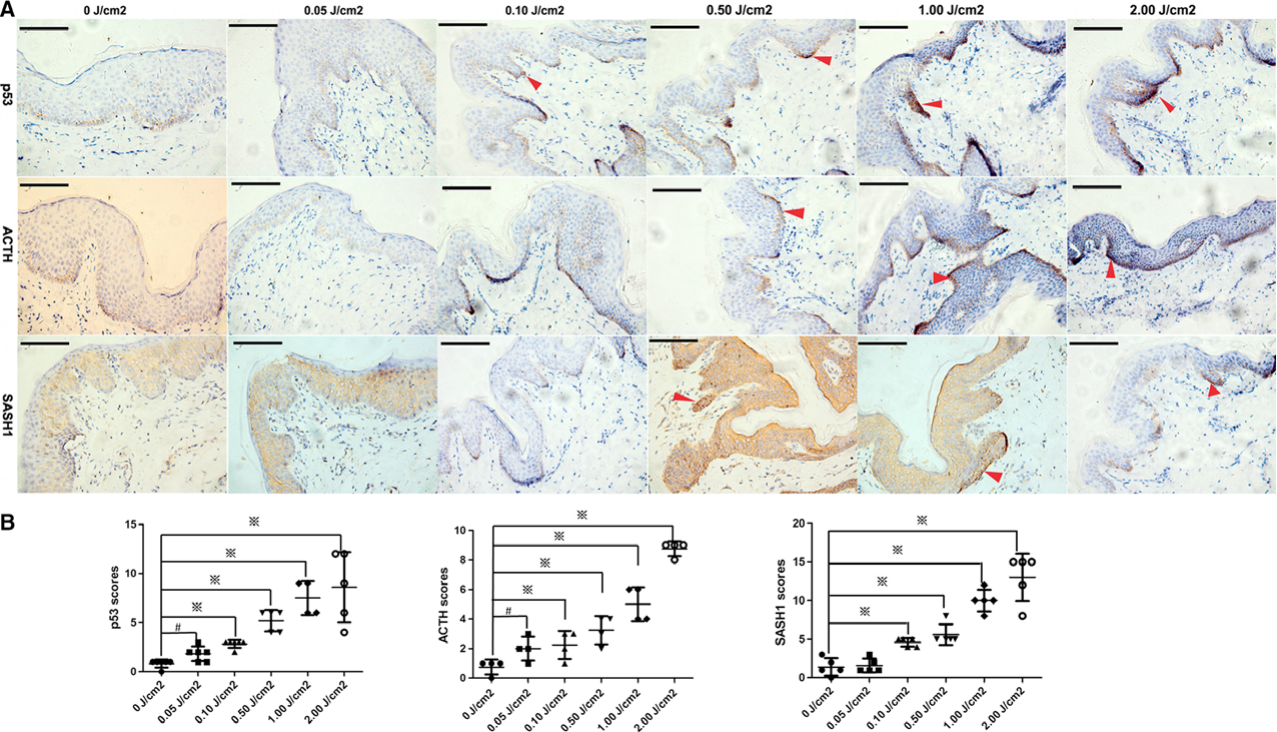
Upon UV irradiation, POMC(ACTH) and SASH1 are induced physiologically by p53. **(A)** Immunohistochemical staining of p53, POMC and SASH1 in human foreskin epithelial tissues showed that the p53 activation by UV irradiation induced up‐regulation of POMC (ACTH) and SASH1. Inductions of p53 and ACTH started at 0.5 J/cm^2^ dose and reached the maximum at 2.0 J/cm^2^. SASH1 induction started at 0.5 J/cm^2^ dose and reached the maximum at 1.0 J/cm^2^ and, however, decreased at 2.0 J/cm^2^. Scale bar: 10 μm. Original magnification: ×400. Red arrows indicate the representative positive cells of p53, ACTH and SASH1. (**B**) 4–6 visual fields in each section of 0, 0.05, 0.10, 0.50, 1.00 and 2.00 J/cm^2^ dose of irradiation were photographed. The staining intensity and percentage of positive cells were calculated and scored in sections of each UV irradiation doses. The total scores of each visual fields were first analysed using the homogeneity of variance test, followed by the change of variable test. Statistical significance was determined by a one‐factor analysis of variance (anova) using LSD on SPSS version 16.0 to generate the required p‐values. The scores were showed as the mean ± S.D. GraphPad Prism 5 was used to plot cartograms. # indicates *P* < 0.05, ※ denotes *P* < 0.01. SASH1: SAM and SH3 domain containing 1.

UV‐mediated induction of POMC‐MSH signal cascade is directly regulated by p53 because that p53 stimulates the POMC promoter in response to UV 13. And as SASH1 is identified to be responsive to POMC, the ligand of MC1R signal pathway, we intend to identify whether SASH1 is responsive to p53 activation *in vivo*. Fresh discarded normal human foreskin specimens were pulled expansion, spread and exposed to gradually increased UV irradiation and stained for the histological analyses of p53, ACTH and SASH1. Immunohistochemical (IHC) analyses revealed p53 is rapidly induced in basal layers at the 0.10 J/cm^2^ dose of UV irradiation. The induction of SASH1 and ACTH at 0.50 J/cm^2^ dose of UV irradiation in melanocytes is induced by UVB irradiation (Fig. 2). Taken together, these results indicate that SASH1 is induced by p53/POMC signal cascade.

### SASH1 binds with MAP2K2, and SASH1 mutations promote binding between SASH1 and MAP2K2

To investigate which pathway that SASH1 is involved in to mediate melanogenesis, pull‐down assay, nanoflow LC‐MS/MS and bioinformatic analyses were performed to investigate the binding partners that SASH1 is associated with in A375 stable cells.

**Figure 3:**
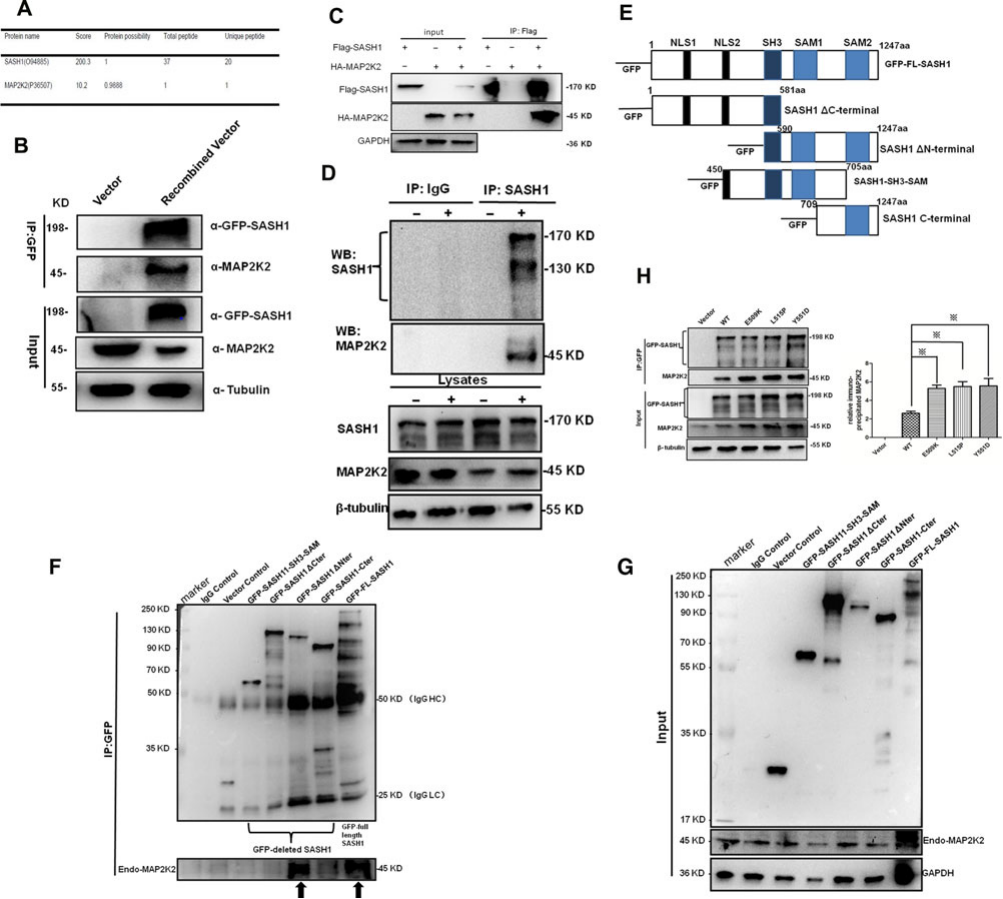
SASH1 interacts with MAP2K2, and SASH1 mutations promote enhanced binding between SASH1 and MAP2K2. **(A)** MAP2K2 showed high possibility to interact with SASH1 as identified by LC‐MS/MS analysis. (**B**) The associations between GFP‐SASH1 and endogenous MAP2K2 were identified by IP‐WB analysis in HEK‐293 cells. HEK‐293T cells were transfected with the pEGFP‐C3‐SASH1 vectors. At 36 hrs after transfection, GFP‐SASH1 was immunoprecipitated (IP) and the associated MAP2K2 was detected by Western blot analyses using MAP2K2 antibody. (**C**) HA‐MAP2K2 is associated with Flag‐SASH1. HEK‐293T cells were cotransfected with the pcDNA3.0‐HA‐MAP2K2 and pBABE‐puro‐Flag‐SASH1 vectors. At 36 hrs after transfection, Flag‐SASH1 was immunoprecipitated, and the associated HA‐MAP2K2 was detected by Western blot analyses using an anti‐HA antibody. (**D**) Endogenous SASH1 is associated with endogenous MAP2K2 in melanocytes. 90% of cell density of NHEMs after 24‐hrs culture was lysed and immunoprecipitated with SASH1 antibody, and the associated immunoprecipitated endogenous MAP2K2 was analysed by Western blot. (**E**) Schematic view of SASH1 deletion constructs. SASH1 contains two predicted nuclear localization signals (NLS1 and NLS2), the conserved Src‐homology 3 domain (SH3) and the two sterile alpha‐motifs (SAM1 and SAM2). Amino acid residues of the SASH1 coding sequence are indicated. Four deleted SASH1 fragments were cloned to pEGFP‐C3 vector and expressed as GFP‐deleted SASH1 fusion proteins. (**F**) and (**G**) C‐terminal domain (590aa‐1247aa) of SASH1 binds to MAP2K2. Four deleted SASH1 fragments including SH3‐SAM construct, ΔC‐terminal construct, ΔN‐terminal construct and C‐terminal construct and SASH1 full length were cloned into pEGFP‐C3 vector and transfected HEK‐293T cells. After 36‐hrs transfection, cells were lysed and immunoprecipitated with GFP antibody, and the associated endogenous MAP2K2 was detected by Western blot analyses using MAP2K2 antibody. As compared with GFP‐full‐length SASH1, C‐terminal domain (590aa‐1247aa) of SASH1 was showed to be associated with endogenous MAP2K2 (indicated by black arrows). (**H**) SASH1 mutations enhanced the associations between SASH1 and MAP2K2. Wild‐type and mutant SASH1‐pEGFP‐C3 were transfected to HEK‐293T cells. At 36 hrs after transfection, transfected cells were lysed and subjected to immunoprecipitation with GFP antibody and the associated MAP2K2 was analysed by Western blot. Left panel: Equal amounts of protein lysates or immunoprecipitates were resolved for Western blotting or immunoprecipitation with anti‐GFP or anti‐MAP2K2 antibody. Right panel: endogenous MAP2K2 which was immunoprecipitated by GFP‐SASH1 was quantified. ※ *P* < 0.01 compared to WT (wild‐type) groups. NHEMs: normal human epithelial melanocytes; SASH1: SAM and SH3 domain containing 1.

Nanoflow LC‐MS/mass spectrometry and bioinformatics analyses indicated that SASH1 may be involved in three pathways involved in or mediating melanin biosynthesis: the insulin/IGF pathway, the mitogen‐activated protein kinase kinase(MAPK)/MAP kinase cascade and the Ras pathway (Table S1). Obviously, MAP2K2 (P36507), a dual‐specificity protein kinase that belongs to the MAP kinase kinase family 18, was shown to be involved in ten pathways including MAPK cascade. And MAP2K2 was showed to have a certain possibility to bind with SASH1 (Fig. 3A and Table S2). Additionally, SASH1 was showed to activate NF‐kB and MAPKs through TLR4 in human endothelial cells to increase production of proinflammatory cytokines 19. To identify the endogenous protein associations between SASH1 and MAP2K2, immunoprecipitation–Western blot (IP‐WB) was performed to identify the associations between SASH1 and MAP2K2. As shown in Figure 3B and C, exogenous SASH1 (GFP‐SASH1 or Flag‐SASH1) is associated with endogenous MAP2K2 or exogenous MAP2K2 (HA‐MAP2K2). And immunoprecipitation with SASH polyclone antibody was performed. As demonstrated in Figure 3D, endogenous SASH1 was showed to immunoprecipitate with endogenous MAP2K2 in NHEMs. We further identify the binding domain of SASH1 to MAP2K2. Deleted SASH1 constructs were created and transfected in HEK‐293T cells and immunoprecipitated with GFP antibody. As compared with the binding of SASH1 full‐length fragment to MAP2K2, SH3‐SAM domain of SASH1 was showed to bind to MAP2K2 (Fig. 3E and F). So, it is believed that SASH1 is associated with MAP2K2 through its SH3‐SAM domain corresponding to the Sly‐homology domain. We also assessed the effects of SASH1 mutations on MAP2K2. We also further analysed the effects of SASH1 mutations on SASH1‐MAP2K2 interaction. IP‐WB analyses indicated that mutated SASH1s showed strong binding ability to MAP2K2 (Fig. 3G).

### SASH1 mutations regulate ERK1/2/CREB cascade

**Figure 4:**
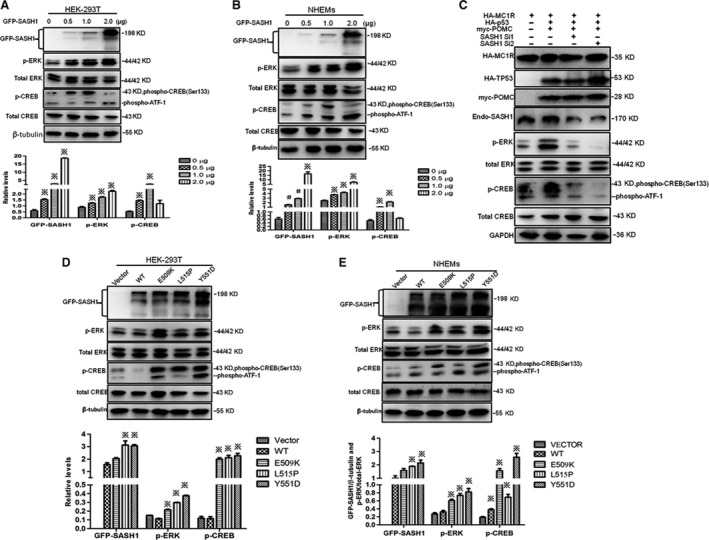
Phospho‐ERK1/2 and phospho‐CREB levels are induced by SASH1 overexpression and SASH1 mutations *in vitro*. (**A**) and (**B**) Phosphorylation levels of ERK1/2 and CREB were induced by SASH1. Different doses of SASH1‐pEGFP‐C3 or SASH1‐pCDH‐EF1‐MCS‐T2A‐copGFP vectors were transfected or infected into HEK‐293T or NHEMs. At 48 hrs after transfection or 48 hrs after infection, cells were lysed and analysed by Western blot along with β‐tubulin as a loading control. Top panels: Western blot results using specific antibodies. Bottom panels: relative protein levels of SASH1 and phospho‐ERK and phospho‐CREB were determined by Western blot in three independent experiments. ※ *P* < 0.01 compared to 0 μg groups. #*P* < 0.05 compared to 0 μg groups. (**C**) SASH1 is necessary for the activation of ERK1/2 and CREB. HEK‐293T cells were transfected with HA‐p53,myc‐POMC and HA‐MC1R,respectively, according to different manners of combination. After 24 hrs after transfection,two specific pairs of SASH1 siRNAs were introduced into the transfected HEK‐293T cells. After 36 hrs after transfection, cells were lysed and subjected to immunoblotting with GAPDH as loading control. (**D**) and (**E**) SASH1 mutations trigger phosphorylation levels of ERK1/2 and CREB in HEK‐293T cells and NHEMs. Wild‐type and mutated exogenous SASH1 were transfected or infected into HEK‐293T cells and NHEMs. At 36 hrs after transfection, cells were lysed and subjected into Western blot analysis along with β‐tubulin as loading control. Relative protein levels of SASH1, phospho‐ERK and phosphor‐CREB (bottom panels) were determined by Western blot (top panels) in three independent experiments. ※ *P* < 0.01 compared to WT groups. NHEMs: normal human epithelial melanocytes; SASH1: SAM and SH3 domain containing 1.

**Figure 5:**
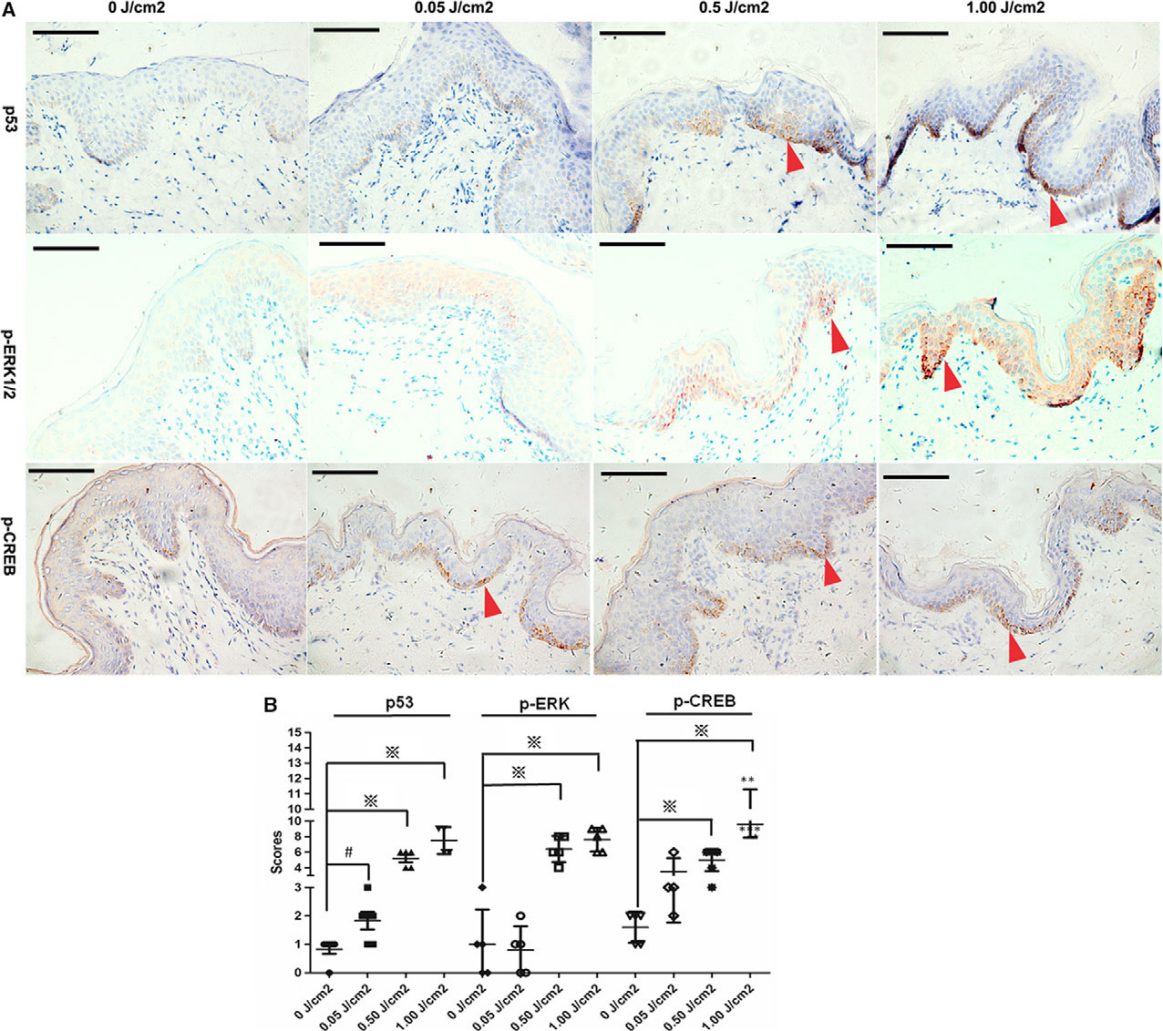
Phosphorylation levels of ERK1/2 and CREB are mediated by p53 induction upon UV irradiation. (**A**) Activation of P53/POMC/SASH1 cascade by UV irradiation induced enhanced phospho‐ERK1/2 and phosphor‐CREB levels in human foreskin epithelial tissues exposed at different doses of UV intensity. Original magnification: ×400 and scale bar: 10 μm. The induction of phospho‐ERK1/2 and phosphor‐CREB started at 0.5 J/cm^2^ and reached the maximum at 1.0 J/cm^2^. Red arrows indicated the representative positive cells of p53, phospho‐ERK1/2 and phospho‐CREB. (**B**) 4–7 visual fields in each section of 0, 0.05, 0.50 and 1.00 J/cm^2^ dose of irradiation were photographed. The staining intensity and percentage of p53‐, p‐ERK1/2‐ and p‐CREB‐positive cells were calculated and scored in sections of each UV irradiation doses. Statistical significance of the scores was determined using one‐way anova with LSD correction on SPSS version 16.0 to generate the required p‐values and represented as the mean ± S.D. Cartograms were plotted using GraphPad Prism 5. # indicates *P* < 0.05, ※ denotes *P* < 0.01. SASH1: SAM and SH3 domain containing 1.

**Figure 6:**
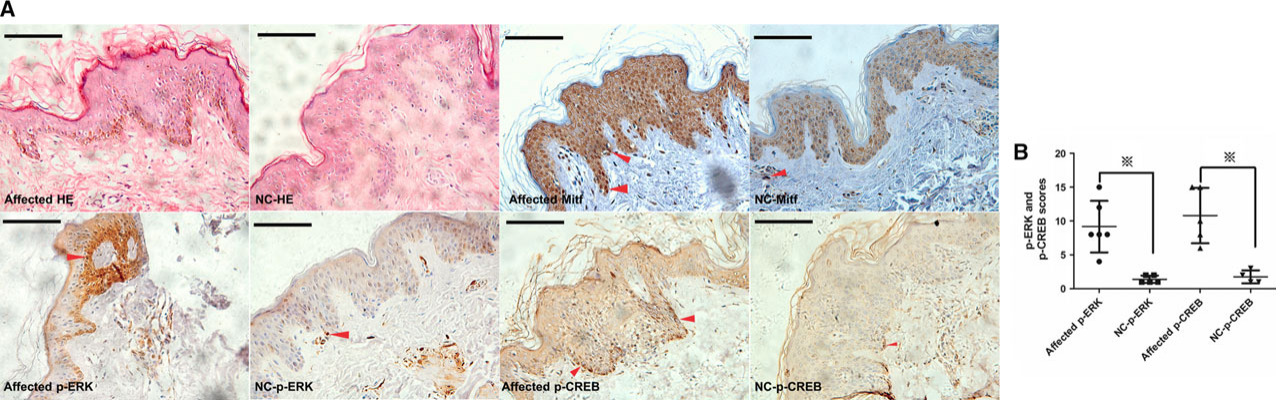
Phosphorylation levels of ERK1/2 and CREB are induced by SASH1 mutations. (**A**) IHC analysis demonstrated that more Mitf‐positive epithelial cells in different epithelial layers in the affected tissues with Y551D‐SASH1 mutation were induced by as compared to those in normal controls (NC). More phospho‐ERK1/2 and CREB in cytoplasm as well as more phospho‐ERK1/2 in nucleus were induced by Y551D‐SASH1 mutation in the affected epithelial layers as compared to those in normal controls. Original magnification: ×400 and scale bar: 10 μm. Red arrows showed the representative positive cells of p53, phospho‐ERK1/2 and phospho‐CREB. (**B**) 4–6 visual fields in each section of affected and normal control individuals were photographed. The staining intensity and percentage of p‐ERK1/2‐ and p‐CREB‐positive cells were evaluated and calculated. Statistical significance of the scores was determined using one‐way anova with LSD correction on SPSS version 16.0 and showed as the mean ± S.D. Cartograms were plotted using GraphPad Prism 5. # indicates *P* < 0.05, ※ denotes *P* < 0.01. SASH1: SAM and SH3 domain containing 1.

We further assessed the effects of SASH1 on the phosphorylation of ERK1/2 downstreams of MAP2K2 and phosphorylation levels of CREB. Gradually increasing doses of exogenous SASH1 were firstly introduced to NHEMs and HEK‐293T cells. Immunoblot analyses showed that SASH1 increased phosphorylation levels of ERK1/2 and CREB (Fig. 4A and B).We also assess the effects of SASH1 silencing on phosphorylation levels of ERK1/2 and CREB. Immunoblotting showed that SASH1 knockdown down‐regulated the phosphorylation levels of ERK1/2 and CREB (Fig. 4C). Additionally, wild‐type and mutant SASH1 were introduced into HEK‐293T cells and NHEMs to assess the effects of SASH1 mutations on phospho‐ERK1/2 and phospho‐CREB. SASH1 mutations increased phosphorylation levels of ERK1/2 and CREB in HEK‐293T cells and NHEMs (Fig. 4D and E). Meanwhile, the effects of p53 activation upon UV irradiation and SASH1 mutation on phosphorylation of ERK1/2 and CREB were also assessed *in vivo*. The fresh human foreskin tissues from a 14‐year‐old boy exposed at different doses of UVB intensity were used for immunohistochemistry analysis. IHC analyses demonstrated that p53 activation caused by UV irradiation induced increase in phospho‐ERK1/2 and phospho‐CREB levels at 0.5 J/cm^2^ dose (Fig. 5). IHC analyses also showed that Y551D mutation enhanced cytoplasmic and nuclear expression of phosphorylated ERK1/2 and phospho‐CREB in the affected epithelial layers as compared to those of normal control (NC) epithelial layers (Fig. 6). Mitf antibody was used to mark melanocytes of affected epithelial layers, and phospho‐ERK positive‐ and phospho‐CREB‐positive epithelial cells are melanocytes.

### Melanogenesis is induced by p53‐POMC‐SASH1 cascade

**Figure 7:**
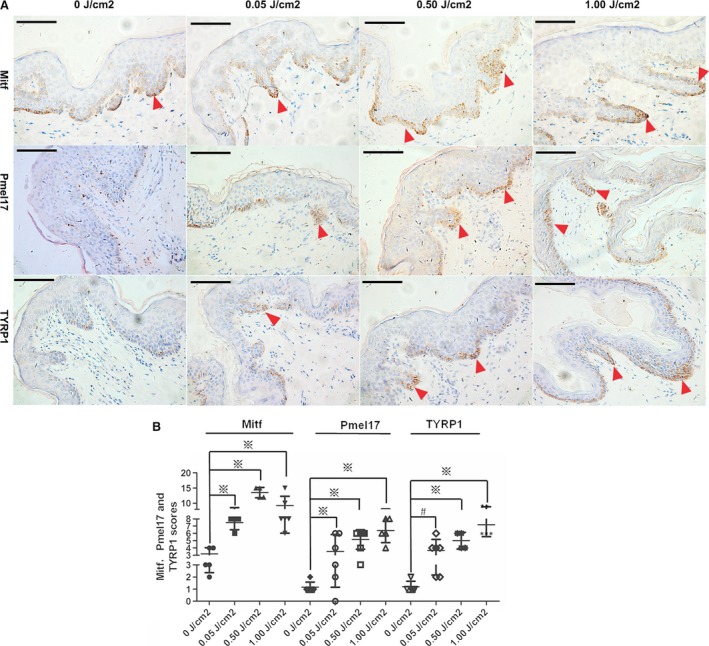
Upon UV irradiation, MITF, Pmel17 and TYRP1 were induced by p53 activation. (**A**) IHC analyses of human foreskin epithelial tissues from a 14‐year‐old boy after UV irradiation indicated that MITF induction by UV irradiation started at 0.05 J/cm^2^ dose and reached the maximum at 0.5 J/cm^2^ and decreased at 1.00 J/cm^2^. However, the induction of Pmel17 and TYRP1 started at 0.05 J/cm^2^ dose and reached the maximum at 1.00 J/cm^2^. Original magnification: ×200 and scale bar: 10 μm. Red arrows indicate the representative positive cells of Mitf, Pmel17 and TYRP1. (**B**) 4–6 visual fields in each section of 0, 0.05, 0.50 and 1.00 J/cm^2^ dose of irradiation were photographed. The staining intensity and percentage of Mitf‐, Pmel17‐ and TYRP1‐positive cells were evaluated and calculated in sections of each UV irradiation doses. Statistical significance of the scores was analysed using one‐way anova with LSD correction and showed as the mean ± S.D. Cartograms were plotted using GraphPad Prism 5. # indicates *P* < 0.05, ※ denotes *P* < 0.01.

We also investigated whether melanogenesis‐specific molecules were responsive to activation of p53‐POMC‐SASH1 cascade. IHC analyses of Mitf, Pmel17 and TYRP1 revealed that the inducement of Mitf started at the 0.05 J/cm^2^ dose of UV irradiation in basal layers, reached the maximum at 0.5 J/cm^2^ dose and decreased at 1.00 J/cm^2^ dose. However, the induction of Pmel17 and TYRP1 started at 0.05 J/cm^2^ dose and reached the maximum at 1.00 J/cm^2^ (Fig. 7). IHC analyses of SILV and tyrosinase also revealed that SILV was induced at 0.05 J/cm^2^ dose of UV irradiation and reached the maximum at 1.0 J/cm^2^ dose. However, no obvious inducement of tyrosinase was observed (Fig. S2).

### Mutated SASH1s are induced by p53, and hyperpigmentation is induced by SASH1 mutations

**Figure 8:**
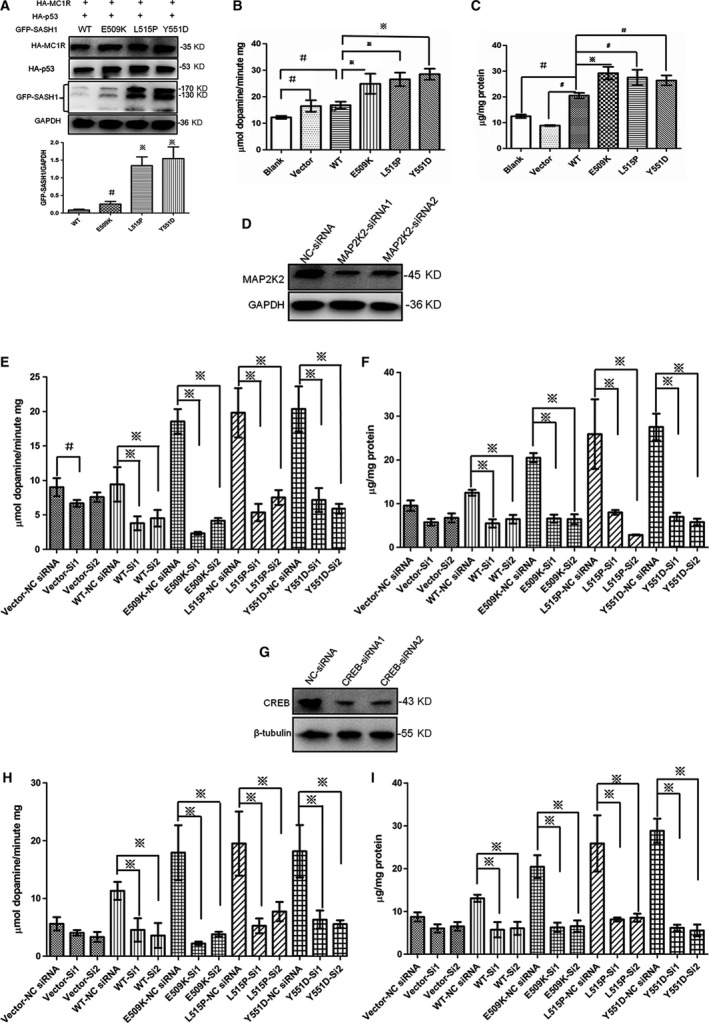
Elevated melanin secretion and increased tyrosinase activity were induced by SASH1 mutations, however, eliminated by silencing MAP2K2 and CREB. (**A**) Mutated exogenous SASH1 was more induced than wild‐type of exogenous SASH1 by p53 in HEK‐293T cells. 1 μg of HA‐p53 plasmid and 1 μg of GFP‐SASH1 (wild‐type and mutated) were cotransfected into HEK‐293T cells pre‐transfected with HA‐MC1R plasmid. Cells were lysed and subjected into Western blot analysis along with GAPDH as loading control. Relative protein levels of endogenous SASH1 (bottom panels) were determined by Western blot (top panels) in three independent experiments. ※ *P* < 0.01 compared to 0 μg groups. (**B)** and (**C**) SASH1 mutations increased the activity of tyrosinase (B) and resulted in elevated melanin secretion (**C**) in NHEMs. NHEMs were infected with wild‐type and mutant SASH1 in 10‐cm dish and cultured in FBS‐free M2 medium. At 48 hrs after infection, cells were washed gently and lysed with IP‐WB lysis buffer. The tyrosinase activity analysis and melanin content assay were performed as per the instruction of manufacturer and determined using one‐way anova with LSD correction on SPSS version 16.0 and showed as the mean ± S.D. Cartograms were plotted using GraphPad Prism 5. ※ denotes *P* < 0.01. # denotes *P* < 0.05. (**D**) MAP2K2 was silenced by two specific MAP2K2 siRNAs efficiently as indicated by Western blot of NHEMs. (**E**) MAP2K2 silencing by its specific siRNAs induced decreased tyrosinase activity in NHEMs transiently transfected with wild‐type and mutant SASH1 genes as well as empty pEGFP‐C3 vector. The tyrosinase activity was determined using one‐way anova with LSD correction on SPSS version 16.0 and showed as the mean ± S.D. ※ denotes *P* < 0.01. # denotes *P* < 0.05. (**F**) MAP2K2 silencing by its specific siRNAs decreased melanin secretion in NHEMs transiently transfected with wild‐type and mutant SASH1 genes as well as empty pEGFP‐C3 vector. Melanin content was determined using one‐way anova with LSD correction and showed as the mean ± S.D. (**G**) Two specific CREB siRNAs could efficiently down‐regulate CREB expression which was silenced as indicated by Western blot of NHEMs. (**H**) CREB silencing induced decrease in tyrosinase activity in NHEMs. (**I**) CREB deletion by its specific siRNAs caused decreased melanin secretion in NHEMs. Tyrosinase activity and melanin content were determined using one‐way anova with LSD correction and showed as the mean ± S.D. ※ denotes *P* < 0.01. NHEMs: normal human epithelial melanocytes; SASH1: SAM and SH3 domain containing 1.

Because the ERK1/2 signalling pathway is identified to be responsive to p53‐POMC‐SASH1 cascade, and the ERK1/2 signalling pathway mediates melanogenesis 10, 11, we sought to address whether mutated SASH1s are induced by p53 and mutations in SASH1 promote biosynthesis of melanogenesis‐specific molecules. Our previous study 9 showed that SASH1 mutations promote the expression of exogenous p53 and more endogenous p53 was induced by Y551D‐SASH1 mutation. Hence, we intend to identify whether p53 could promote mutated SASH1 expression. Our study indicated that mutated SASH1s were more subjected to be induced than wild‐type of SASH1 by p53 in the presence of exogenous MC1R in HEK‐293T cells (Fig. 8A). So, we deduced that p53 activation plays a pivotal role in the pathogenesis process of this dyschromatosis disorder and activation of p53‐POMC‐SASH1 cascade triggers the formation of hyperpigmentation phenotype.

Because A375 cells cannot synthesize melanin (data not showed), we used NHEMs to verify the effects of SASH1 on melanin synthesis. *In vitro* analysis indicated that increased melanin biosynthesis is induced by SASH1 mutations (Fig. 8C). We further tested the enzymatic activity of the rate‐limiting melanogenic enzyme tyrosinase. As expected, we found that SASH1 overexpression and mutations in the SASH1 gene induced increased tyrosinase activity in NHEMs (Fig. 8B). To identify the regulation of SASH1/MAP2K2/ERK1/2/CREB cascade to melanogenesis, in NHEMs transiently expressing mutant and wild‐type SASH1, endogenous MAP2K2 and endogenous CREB were silenced by their specific siRNA. Deletion of MAP2K2 and CREB could decrease tyrosinase activity and melanin production (Fig. 8D–I).

**Figure 9:**
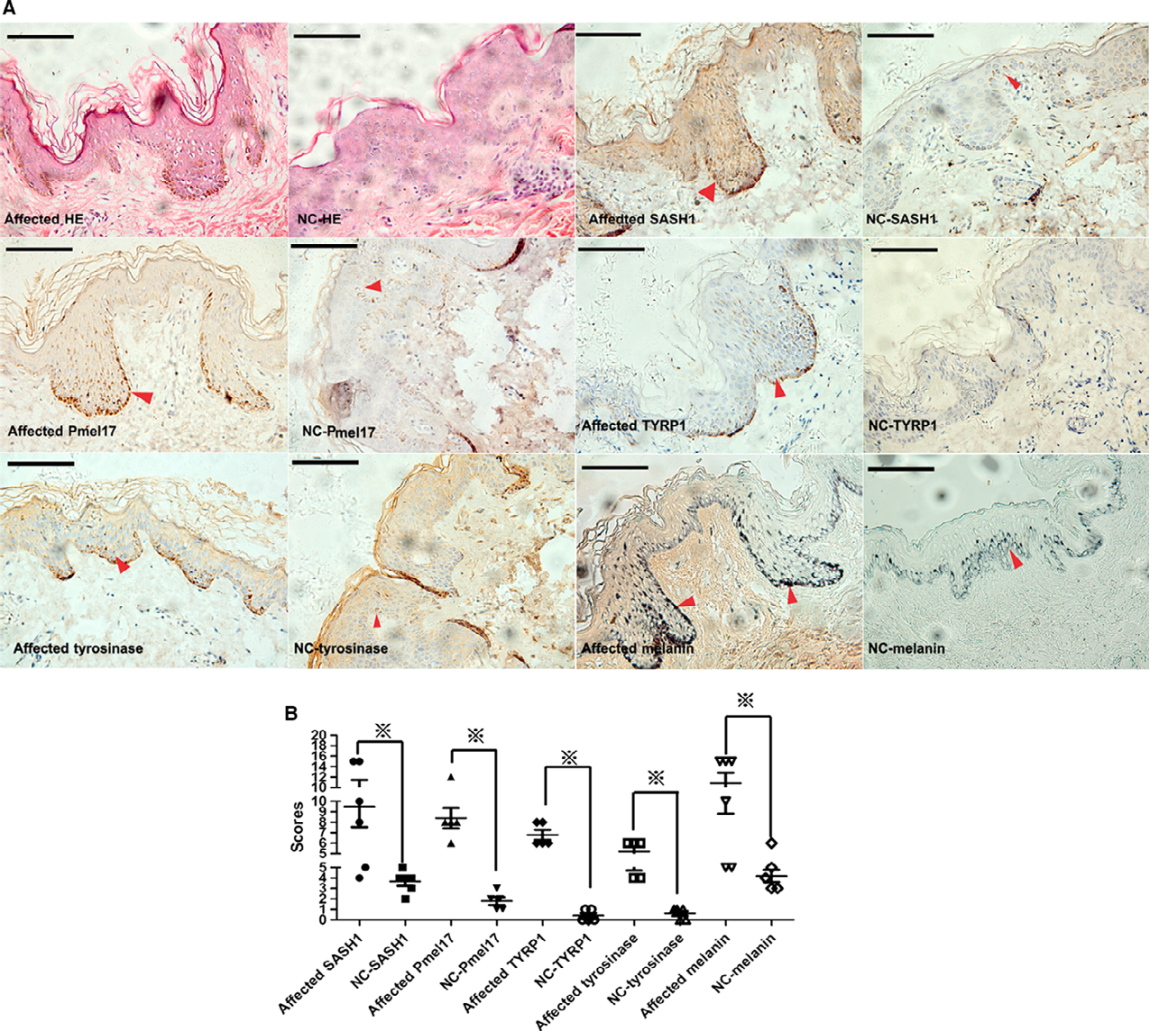
Melanogenic components were induced by SASH1 mutations. (**A**) IHC analysis demonstrated that in the affected epithelial tissues with Y551D mutation,heterogeneous expression of SASH1 was exhibited. Heterogeneous expression of Pmel17, tyrosinase, Rab27a and TYRP1 in affected epithelial layers was induced by SASH1 Y551D mutation, which caused heterogeneous distribution and more synthesized melanin in different affected layers. Original magnification: ×400 and scale bar: 10 μm. Red arrows denote the representative positive cells of SASH1, Pmel17, TYRP1, tyrosinase and melanin. (**B**) 5–6 visual fields in each section of affected and normal control individuals were photographed. The staining intensity and percentage of SASH1‐, Pmel17‐, TYRP1‐, tyrosinase‐ and melanin‐positive cells were calculated and scored. Statistical significance of the scores was determined using one‐way anova with LSD correction and represented as the mean ± S.D. Cartograms were plotted using GraphPad Prism 5. ※ denotes *P* < 0.01. SASH1: SAM and SH3 domain containing 1.

Immunohistochemical (IHC) analyses also revealed heterogeneous expression of SASH1, Pmel17, tyrosinase, TYRP1 and melanin in affected epithelial tissues with Y551D mutation. High levels of SASH1, Pmel17, tyrosinase and TYRP1 expression were found in some regions of affected epithelial layers in Y551D‐affected individuals (Fig. 9). Excessive melanin secretion was observed in both basal and superbasal layers of affected tissues (Fig. 9).

**Figure 10:**
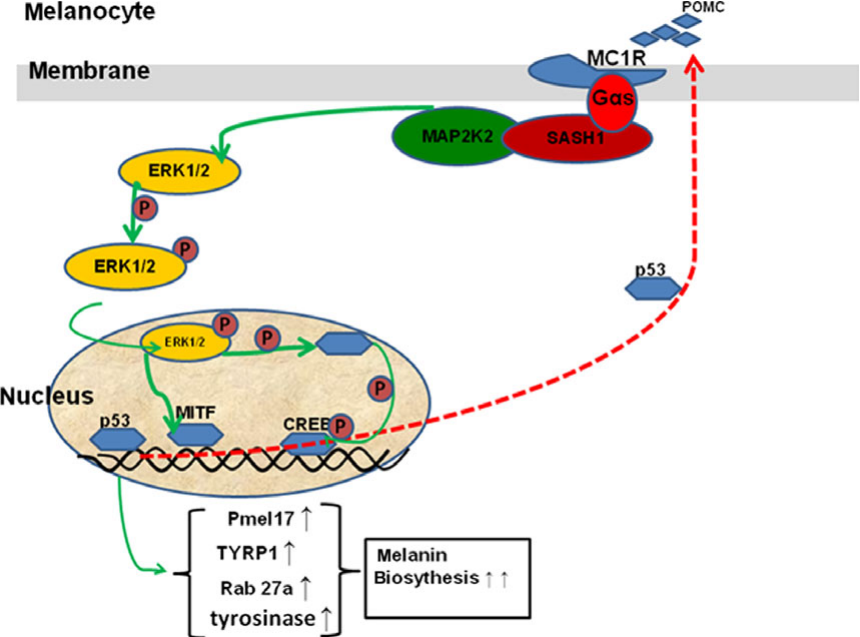
A putative model to demonstrate that p53 regulates ERK1/2/CREB cascade *via* a novel SASH1/MAP2K2 crosstalk to cause hyperpigmentation phenotype in melanocytes. SASH1: SAM and SH3 domain containing 1.

Taken above, we can conclude that a novel p53/POMC signal cascade transactivates ERK1/2/CREB signalling pathway through a novel SASH1/MAP2K2 crosstalk to mediate melanin biosynthesis (Fig. 10). Mutated SASH1 serves as a molecular motor to activate the p53/POMC signal cascade, increasing phosphorylated ERK1/2 and CREB to induce pathological hyperpigmentation.

## Discussion

Dendrite extension and melanin production or secretion are manifestations of melanogenesis that depend on elevated intracellular cAMP levels and are tightly regulated at numerous steps. The cAMP‐dependent activation of Ras protein leading to ERK activation independent of protein kinase A (PKA) and Rap1 is an important part of the molecular underpinnings of melanogenesis 20. Furthermore, recent study had showed that SASH1 is showed to be involved in autosomal dominant lentiginous 21 and autosomal‐recessive SASH1 variants are associated with a new genodermatosis with a pigmentation defects, palmoplantar keratoderma 22.Our work herein suggests that hyperpigmentation phenotype of dyschromatosis universalis hereditaria is attributed to three novel SASH1 point mutations. SASH1 could be a novel exchange factor, which is mediated by p53‐POMC‐MC1R‐cascade, and regulates MAP2K2/ERK1/2/CREB signal pathway to induce melanogenesis process. SASH1 mutations intensify activation of p53‐POMC‐MC1R‐SASH1 cascade and binds with MAP2K2 to mediate ERK1/2 signal pathway, leading to hyperpigmentation of this dyschromatosis disorder.

P53 directly mediates UV induction of POMC/MSH in skin through stimulation of the POMC promoter in response to UV and triggers UV‐independent pathologic pigmentation that could mimic the tanning response 13. Such agonists as a‐melanocyte‐stimulating hormone (α‐MSH) or adrenocorticotropic hormone stimulate MC1R to induce increased melanocyte proliferation and dendricity 19, 23, melanosome transfer 24 and an elevated ratio of the darker eumelanin pigment over the red/yellow pheomelanin pigment 25. Our findings document SASH1 is induced by p53/POMC cascade (Figs 1 and 4). POMC stimulation induces elevated SASH1 expression (Fig. 1C and D). Hence, SASH1 serves as a novel partner to involve in p53‐POMC‐MC1R cascade.

The role of G protein subunit‐coupled receptors in cell proliferation is rather complex, although the activation of Gs and PKA is generally associated with growth inhibition 26. This study provides new insight into the GPCR signalling pathway. SASH1 can respond to MC1R stimulation by POMC. PKA‐independent and cAMP‐dependent pathways have been linked to the regulation of melanogenesis, including the phosphatidylinositol 3‐kinase and Ras/Erk pathways 20. Contrary to many other cell types, B‐Raf, mitogen‐activated protein kinase/extracellular signal‐regulated kinase (MEK) and ERK have been shown to be activated through Ras induction by elevated cAMP in B16 melanoma cells 1, 10, 20, 27. Ras/ERK activation in these cells has a short‐term stimulatory effect, whereby ERK phosphorylates Mitf, leading to increased transcriptional activity of the tyrosinase promoter 10, 28, 29. MAP2K2 was initially indentified as an ERK activator from growth factor‐treated cells and phosphorylates MAPK1/ERK2 cascade and MAPK2/ERK3 cascade 18. A novel c.383C̀‐**>**A transversion in exon 3 of MAP2K2 and 19p13.3 microdeletion involving the mitogen‐activated protein kinase kinase 2 gene (MAP2K2) were reported to cause the cardio‐facio‐cutaneous (CFC) syndrome 30, 31. In this study, MAP2K2 is associated with SASH1 to form a novel SASH1/MAP2K2 crosstalk to regulate phosphorylation level of ERK1/2 and CREB, which suggests that SASH1 is involved in MEK/ERK signal cascade (Fig. 3).

Caveolin‐1‐mediated activation of ERK/CREB signalling may be involved in neuronal transmission pathways implicated in chronic neuropathic pain modulation 32. Activation of phospho‐ERK may partly be accomplished by CREB‐dependent gene expression in chronic constrictive injury in rats 33. Blocking of transient receptor potential canonical (TRPC) channels resulted in suppression of PDGF‐induced Pyk2/ERK/CREB activation, but not PI3K/Akt pathway 34. BMP‐6 plays a crucial role in survival of cerebellar granule cells *via* the noncanonical MEK/ERK/CREB pathway^35^. One of the main targets of the MEK/ERK pathway is CREB. CREB activation has been implicated in synaptic plasticity, learning, and memory and cell survival 36, 37. Our study indicates that the noncanonical MEK/ERK/CREB pathway is mediated by SASH1 (Fig. 4A and B) and SASH1 is necessary for the regulation of ERK/CREB cascade by p53/POMC/MC1R cascade (Fig. 4C). Moreover, SASH1 alleles aggravate the activation when overexpressing in HEK‐293T cells and NHEMs (Fig. 4D and E).

Biosynthesis of melanin, the primary pigment synthesized by mammals, is sequestered within melanosomes, which are unique membrane‐enclosed structures of melanocytes. Consistent with their singular structure and function, melanosomes contain specific resident proteins expressed only in melanocytes and retinal epithelial cells. Most of these characterized proteins are integral membrane proteins, such as tyrosinase, tyrosine‐related protein 1 (TYRP1) and Pmel17 (gp100). Pmel17 is a matrix protein of premelanosomes, whereas TYRP1 is a component of mature melanosomes 38. Pmel17 is first enriched in stage I‐like structures and accumulates to a higher extent in stage II premelanosomes. In contrast, TYRP1 is almost undetectable in stage I structures, and only a small percentage of labelling is observed in stage II. These published results suggest that Pmel17 is a major biogenetic component of the striations 39. Our study suggests that SASH1 point mutations result in increased expression levels of Pmel17, TYRP1 and tyrosinase, leading to melanin production and secretion *in vivo* (Fig. 9). More importantly, our studies demonstrate that p53 regulates the melanogenesis process in a SASH1‐dependant manner, which will underscore the p53 role in the formation of hyperpigmentation phenotype in dyschromatosis.

## Author contributions

DAZ, QHX and LH designed the experiments and analysed the data. DAZ, KW, ZYW, ZSK, HCL, JSM, XZ, FJL, MC, SL, HL and ZYW performed the experiments and analysed the data. YH, JWZ, HYD, HL and BZL provided clinical samples and scientific support. DAZ, QHX and LH wrote and revised the manuscript.

## Conflict of interest

The authors declare no competing financial interests.

## Supporting information


**Fig. S1** NHEMs and HEK‐293T transfected cells had MC1R responsiveness.

**Fig. S2** SILV and tyrosinase were induced by p53 activation upon UV irradiation.

**Table S1** Bioinformatic analysis of pathways that SASH1 may involve in

**Table S2** The peptide sequences of the SASH1 complex identified by SBP‐FLAG–SASH1 affinity purification

**Table S3** Detailed information for each antibody used in this study
