# Supplementary figures and images for: p53 regulates ERK1/2/CREB cascade via a novel SASH1/MAP2K2 crosstalk to induce hyperpigmentation
Source: J Cell Mol Med. 2017 Apr 6;21(10):2465–80. doi: 10.1111/jcmm.13168 (PMC5618682; doi:10.1111/jcmm.13168)

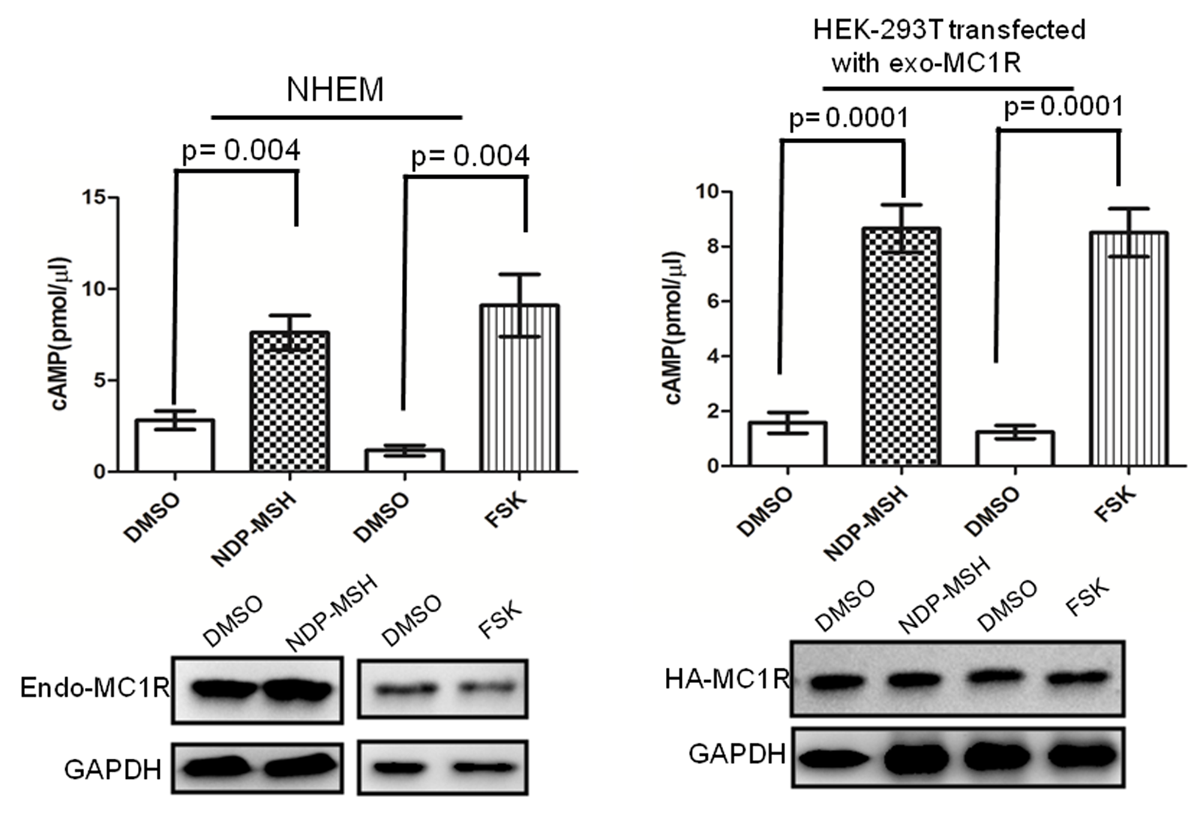

Supplement: Supplementary file 1 — Fig. S1 NHEMs and HEK‐293T transfected cells had MC1R responsiveness. [file JCMM-21-2465-s001.tif]

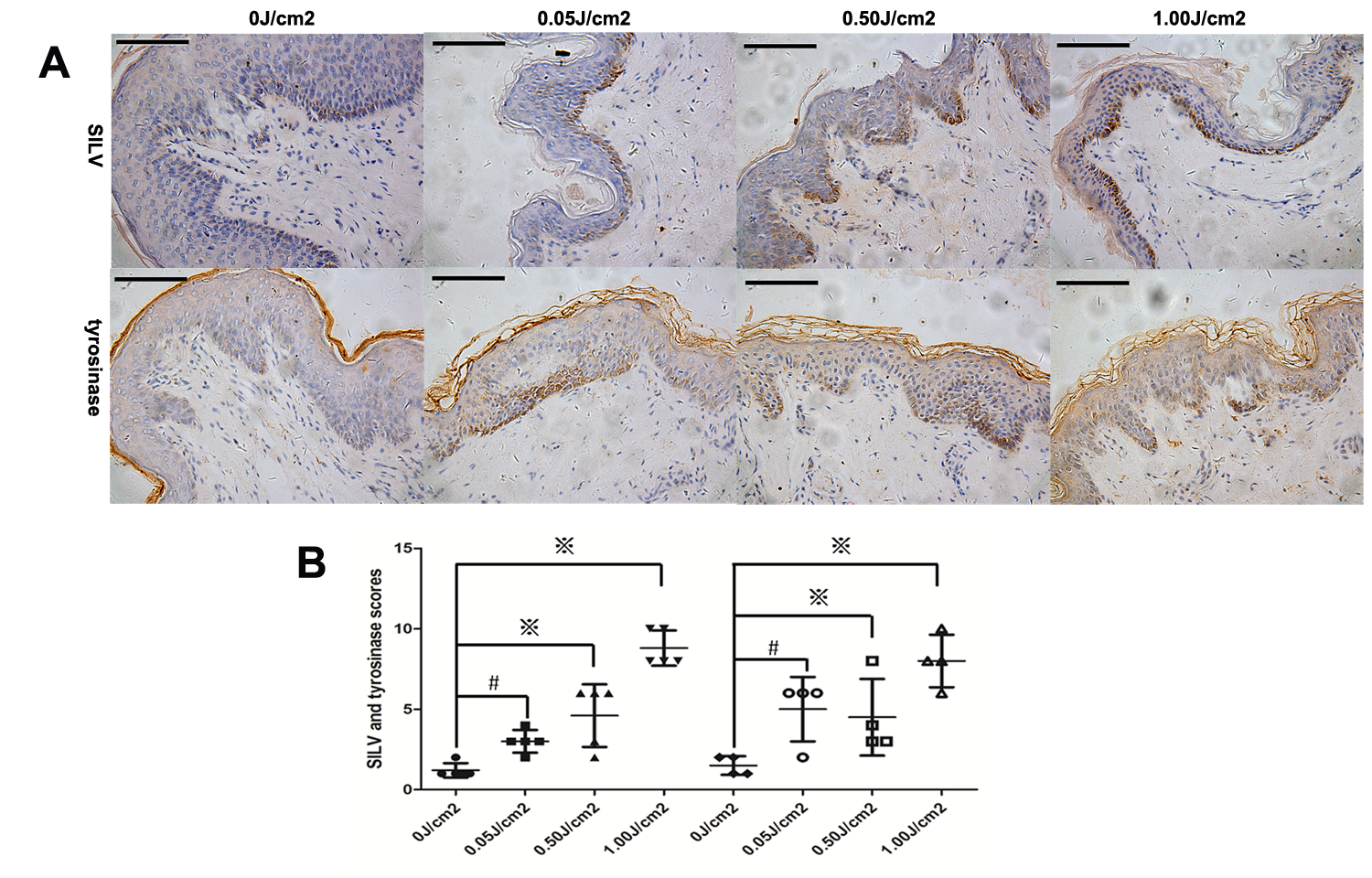

Supplement: Supplementary file 2 — Fig. S2 SILV and tyrosinase were induced by p53 activation upon UV irradiation. [file JCMM-21-2465-s002.tif]
