# Supplementary material for: p53 regulates ERK1/2/CREB cascade via a novel SASH1/MAP2K2 crosstalk to induce hyperpigmentation
Source: J Cell Mol Med. 2017 Apr 6;21(10):2465–80. doi: 10.1111/jcmm.13168 (PMC5618682; doi:10.1111/jcmm.13168)
Supplement: Supplementary file 3 — Table S1 Bioinformatic analysis of pathways that SASH1 may involve in [file JCMM-21-2465-s003.docx]

**Supplementary Table 1 Bioinformatic analysis of pathways that SASH1 may involve in**

|  | Classification criteria |  |  |  |
| --- | --- | --- | --- | --- |
| **go term ID** | pathway | identified proteins number | **percent** | identified proteins |
| P00034  P00046  P00029  P00054  P00018  P00028  P00049  P02746  P05911  P00016  P00021  P00053  P00010  P00019  P00044  P00032  P02776  P00047  P00024  P04393 | **Integrin signalling pathway**  **Oxidative stress response**  **Huntington disease**  **Toll receptor signaling pathway**  **EGF receptor signaling pathway**  **Heterotrimeric G-protein signaling pathway-rod outer segment**  **phototransduction**  **Parkinson disease**  **Heme biosynthesis**  **Angiotensin II-stimulated signaling through G proteins and beta-arrestin**  **Cytoskeletal regulation by Rho GTPase**  **FGF signaling pathway**  **T cell activation**  **B cell activation**  **Endothelin signaling pathway**  **Nicotinic acetylcholine receptor signaling pathway**  **Insulin/IGF pathway-mitogen activated protein kinase kinase/MAP kinase cascade**  **Serine glycine biosynthesis**  **PDGF signaling pathway**  **Glycolysis**  **Ras Pathway** | **2**  **1**  **4**  **1**  **2**  **1**  **1**  **1**  **1**  **3**  **2**  **2**  **2**  **1**  **1**  **1**  **1**  **1**  **1**  **1** | **3.64%**  **1.82%**  **7.27%**  **1.82%**  **3.64%**  **1.82%**  **1.82%**  **1.82%**  **1.82%**  **5.45%**  **3.64%**  **3.64%**  **3.64%**  **1.82%**  **1.82%**  **1.82%**  **1.82%**  **1.82%**  **1.82%**  **1.82%** | **P36507, O43707;**  **P35637;**  **O15484,Q9BUF5,P84085 ,P07437;**  **P36507;**  **P62258, P36507;**  **P62158;**  **P62258;**  **P07814;**  **P36507;**  **P07437,P07737 ,Q9BUF5;**  **P62258, P36507;**  **P36507 ,P62158;**  **P36507, P62158;**  **P36507;**  **O00159;**  **P36507;**  **O43175;**  **P36507;**  **P00558;**  **P36507.** |
