# Supplementary material for: p53 regulates ERK1/2/CREB cascade via a novel SASH1/MAP2K2 crosstalk to induce hyperpigmentation
Source: J Cell Mol Med. 2017 Apr 6;21(10):2465–80. doi: 10.1111/jcmm.13168 (PMC5618682; doi:10.1111/jcmm.13168)
Supplement: Supplementary file 4 — Table S2 The peptide sequences of the SASH1 complex identified by SBP‐FLAG–SASH1 affinity purification [file JCMM-21-2465-s004.docx]

**Supplementary Table 2** The peptide sequences of the SASH1 complex identified by SBP-FLAG–SASH1 affinity purification

The precipitated proteins from stable A375 cells expressing SBP-FLAG–SASH1 were digested with trypsin. The supernatant was collected, dried and dissolved in 10% (v/v) acetonitrile and 0.8% formic acid solution. The peptides were analysed by LC-MS/MS.

| Protein name | Peptide sequence |
| --- | --- |
| SASH1(O94885) | KKPSTEGGEEHVFENSPVLDER.S  R.AVLLTAVELLQEYDSNSDQSGSQEK.L  KGEDVGYVASEITMSDEERI  RVSQDLEVEKPDASPTSLQLRS  RVHTDFTPSPYDTDSLKI  KLLEEEDLDELNIRD  KLHAEGIDLTEEPYSDKH  KPGAGTSEAFSRL  KPLFFDGSPEKPPEDDSDSLTTSPSSSSLDTWGAG  KMGTFFSYPEEEKA  KMITIEEALARL  R.SLHVGSNNSDPMGKE  SLHVGSNNSDPMGK  ITIEEALAR  MITIEEALARL  RGVDLETLTENKL  IPSQPPPVPAK  TIEEALAR  KYFWQNFR.K  SALYSGVHK |
| MAP2K2（P36507） | PAMAIFELLDYIVNEPPPK. |
|  |  |
