# Supplementary material for: p53 regulates ERK1/2/CREB cascade via a novel SASH1/MAP2K2 crosstalk to induce hyperpigmentation
Source: J Cell Mol Med. 2017 Apr 6;21(10):2465–80. doi: 10.1111/jcmm.13168 (PMC5618682; doi:10.1111/jcmm.13168)
Supplement: Supplementary file 5 — Table S3 Detailed information for each antibody used in this study [file JCMM-21-2465-s005.docx]

Supplementary Table 3 Detailed information for each antibody used in this study

| Antibody Name | Manufacturer (catalogue number) | Species Origin | Antibody application and dilutions | Dilution Liquid for antibody |
| --- | --- | --- | --- | --- |
| DYKDDDDK  Flag-Tag(3B9) | Shanghai abmart(#M20008)  Shanghai Abmart(#M20002) | Mouse mAb^1^  Mouse mAb^1^ | IB^2^(1/2000)  IB^2^(1/2000) | TBS-Tween(0.1%)-BSA(5%)  TBS-Tween(0.1%)-BSA(5%) |
| GFP-Tag(7G9) | Shanghai Genomics(SG4110-25) | Mouse mAb | IB(1/2000) | TBS-Tween(0.1%)-BSA(5%) |
| MYC-Tag(19C2) | Shanghai Genomics(SG4110-25) | Mouse mAb | IB(1/2000) | TBS-Tween(0.1%)-BSA(5%) |
| HA-Tag  P53(7F5) | Shanghai Genomics(SG4110-25)  Cell Signaling Technology (cat:2527S, lot:6) | Mouse mAb  Rabbit mAb | IB(1/2000)  IB(1/2000) | TBS-Tween(0.1%)-BSA(5%)  TBS-Tween(0.1%)-BSA(5%) |
| P53(7F5)  ACTH(7-23)  MC1R  MC1R  SASH1  Phospho-ERK1/2  (Thr202/Tyr204)  Phospho-ERK1/2  (Thr202/Tyr204)  Phospho-CREB  (Ser133)(87G3)  Phospho-CREB  (Ser133)(87G3)  MITF(G174)  Mitf(SPM290)  SILV (Clonc 9G3)  Tyrosinase | Cell Signaling Technology (cat:2527S lot:6)  Beijing Biosynthesis biotechnology(cat:bs-0004R, lot:20909086 )  Beijing Biosynthesis biotechnology(cat:bs-1419R, lot:AD04291810 )  Novus Biologicals(Cat:NBP96040)  Novus(NBP1-26650)  Cell Signaling Technology (#9101)  Cell Signaling Technology (#9101)  Cell Signaling Technology (#9198)  Cell Signaling Technology (#9198)  Bioworld (cat:BS1550  Lot :G22121)  Novus(Lot:NBP2-45160)  ORIGENE(cat:TA500400)  Abcam(ab61294) | Rabbit mAb  Rabbit pAb  Rabbit pAb  Rabbit mAb  Rabbit pAb  Rabblit pAb  Rabblit mAb  Rabblit mAb  Rabblit mAb  Rabblit pAb  Mouse mAb  Rabblit mAb  Rabblit mAb | IHC^3^ (1:500)  IHC(1:200)  IHC(1:200)  IB(1/1000)  IIB(1/1000)  IHC^4^(1/500)  IHC(1:500)  IB(1/2000)  IHC(1/500)  IHC(1/100)  IHC(1/100)  IHC(1/100)  IHC(1/500) | PBS  PBS  PBS  TBS-Tween(0.1%)-BSA(5%)  TBS-Tween(0.1%)-BSA(5%)  PBS  PBS  TBS-Tween(0.1%)-BSA(5%)  PBS  PBS  PBS  PBS |
| SASH1 | Novus(NBP1-26650) | Rabblit pAb^4^ | IB(1/2000) | TBS-Tween(0.1%)-BSA(5%) |
|  |  |  |  |  |
| SASH1 | BETHYL(A302-265A-1) | Rabblit pAb | IB(1/2000) | TBS-Tween(0.1%)-BSA(5%) |
| SASH1 | BETHYL(A302-265A-1) | Rabblit pAb | IHC(1/500) | PBS |
| Rab 27a | Abcam(ab55667) | Mouse mAb | IHC and IF^5^(1/500) | PBS |
| Rab 27a | Novus(H0005873-M01) | Mouse mAb | IB(1/2000) | TBS-Tween(0.1%)-BSA(5%) |
| Tyrosinase | Abcam(ab61294) | Rabblit pAb | IB(1/1000) | TBS-Tween(0.1%)-BSA(5%) |
| ERK1/2 | Cell Signaling Technology (#9102) | Rabblit mAb | IB(1/2000) | TBS-Tween(0.1%)-BSA(5%) |
| CREB (48H2) | Cell Signaling Technology (#9197) | Rabblit mAb | IB(1/2000) | TBS-Tween(0.1%)-BSA(5%) |
| GAPDH(2A8) | Shanghai Abmart (#M20028) | Mouse mAb | IB(1/1000) | TBS-Tween(0.1%)-BSA(5%) |
| TYRP1  TYRP1 | Abcam(Ab3312)  Abcam(Ab3312) | Rabblit mAb  Rabblit mAb | IHC (1/200)  IB(1/500) | PBS  TBS-Tween(0.1%)-BSA(5%) |
| Melanoma gp100(Pmel17)  Melanoma gp100(Pmel17) | Abcam (ab137062)  Abcam (ab137062) | Rabblit mAb  Rabblit mAb | IHC and IF(1/200)  IB(1/500) | PBS  TBS-Tween(0.1%)-BSA(5%) |

1, mAb, monoclonal antibody; 2,IB,immunoblot；3，IHC, immunohistochemistry；4，pAb，polyclonal antibody; 5, IF, immunofluorescence.
