# Supplementary material for: p53 regulates ERK1/2/CREB cascade via a novel SASH1/MAP2K2 crosstalk to induce hyperpigmentation
Source: J Cell Mol Med. 2017 Apr 6;21(10):2465–80. doi: 10.1111/jcmm.13168 (PMC5618682; doi:10.1111/jcmm.13168)
Supplement: Supplementary file 6 [file JCMM-21-2465-s006.docx]

**Fig. S1 NHEMs and HEK-293T transfected cells had MC1R responsiveness**

In order to identify the NHEMs and the HEK-293T cells that we pre-transfected HA-MC1R-Pcnd3.0 have MC1R responsiveness, NHEMs and HEK-293T transfected cells were starved for 8hr and treated with 10^-5^M of NDP-MSH for 30min, respectively. Cells were lysed and subjected to quantification of protein concentration and ELISA to detect the concentration of intracellular cAMP. Intracellular cAMP levels between DMSO groups and NDP-MSH treated groups were compared using 1-way ANOVA with LSD correction and represented as the mean ± SD. Intracellular cAMP levels were induced by the stimulation of NDP-MSH, which indicated that both cells had MC1R responsiveness. Meanwhile, immunoblot showed that the expression of endogenous MC1R and exogenous MC1R.

**Fig. S2 SILV and tyrosinase were induced by p53 activation** **upon UV irradiation**

(**A**) IHC analyses of human foreskin epithelial tissues from a 14 year-old boy after UV irradiation indicated that induction of SILV and tyrosinase by UV irradiation started at 0.05J/cm^2^ dose and reached the maximum at 1.00J/ cm^2^. Original magnification: ×200 and scale bar: 10µm. Red arrows denoted the representative positive cells of SILV and tyrosinase. (**B**) 4～5 visual fields in each section of 0J/cm^2^, 0.05J/cm^2^, 0.50J/cm^2^ and 1.00 J/cm^2^ dose of irradiation were photographed. The staining intensity and percentage of SILV- and tyrosinase-positive cells were evaluated and calculated in sections of each UV irradiation doses. Statistical significance of the scores was determined using 1-way ANOVA with LSD correction and represented as the mean ± SD. Cartograms were plotted using GraphPad Prism 5. # indicates P<0.05, ※ denotes p<0.01.
